# Supplementary material for: Comparative genomics provides new insights into the diversity, physiology, and sexuality of the only industrially exploited tremellomycete: Phaffia rhodozyma
Source: BMC Genomics. 2016 Nov 9;17:901. doi: 10.1186/s12864-016-3244-7 (PMC5103461; doi:10.1186/s12864-016-3244-7)
Supplement: Additional file 6: — List of orphan genes with links to PFAM (related to Additional file 1: Table S1). (ZIP 1428 kb) [file 12864_2016_3244_MOESM6_ESM.zip › BLAST_HTML_FTR/G02123_P.html]

BLAST Search Results


```
BLASTP 2.2.27+


Reference:
Stephen F. Altschul, Thomas L. Madden, Alejandro A. Schäffer,
Jinghui Zhang, Zheng Zhang, Webb Miller, and David J. Lipman (1997),
"Gapped BLAST and PSI-BLAST: a new generation of protein database
search programs", Nucleic Acids Res. 25:3389-3402.


Reference for
composition-based statistics:
Alejandro A. Schäffer, L. Aravind, Thomas L. Madden, Sergei
Shavirin, John L. Spouge, Yuri I. Wolf, Eugene V. Koonin, and
Stephen F. Altschul (2001), "Improving the accuracy of PSI-BLAST
protein database searches with composition-based statistics and
other refinements", Nucleic Acids Res. 29:2994-3005.


Database: nr
           71,551,133 sequences; 26,053,659,533 total letters


Query= G02123_P

Length=394
                                                                      Score     E
Sequences producing significant alignments:                          (Bits)  Value

emb|CED82152.1|  hypothetical protein [Xanthophyllomyces dendrorh...   764    0.0  
emb|CDZ96279.1|  EME1, MMS4 [Xanthophyllomyces dendrorhous]           50.8    0.002
ref|XP_007351565.1|  hypothetical protein AURDEDRAFT_170588 [Auri...  50.1    0.003
gb|KIR87918.1|  crossover junction endonuclease EME1 [Cryptococcu...  48.1    0.010
gb|KIR63608.1|  crossover junction endonuclease EME1 [Cryptococcu...  48.1    0.012
gb|KIR47882.1|  crossover junction endonuclease EME1 [Cryptococcu...  48.1    0.013
gb|KEP53600.1|  ERCC4 domain protein [Rhizoctonia solani 123E]        47.8    0.015
gb|EMS25983.1|  ERCC4 domain containing protein [Rhodosporidium t...  47.8    0.015
gb|EUC67094.1|  ERCC4 domain protein, partial [Rhizoctonia solani...  47.4    0.020
gb|KDN49392.1|  hypothetical protein RSAG8_02094, partial [Rhizoc...  47.4    0.022
gb|EKD04587.1|  vesicle membrane protein (v-SNARE) with acyltrans...  45.4    0.091
gb|KIK71135.1|  hypothetical protein GYMLUDRAFT_235500 [Gymnopus ...  45.4    0.098
gb|KIR54999.1|  crossover junction endonuclease EME1 [Cryptococcu...  45.1    0.12 
ref|XP_003196799.1|  hypothetical protein CGB_K3270C [Cryptococcu...  45.1    0.12 
gb|EJT46446.1|  vesicle membrane protein (v-SNARE) with acyltrans...  45.1    0.14 
gb|KIY35389.1|  crossover junction endonuclease EME1 [Cryptococcu...  44.3    0.17 
ref|XP_001731355.1|  hypothetical protein MGL_1538 [Malassezia gl...  43.9    0.21 
ref|XP_012048427.1|  crossover junction endonuclease EME1 [Crypto...  43.1    0.48 
ref|XP_013240836.1|  hypothetical protein K437DRAFT_276193 [Tille...  41.6    1.5  
ref|XP_013084856.1|  PREDICTED: crossover junction endonuclease M...  40.0    2.6  
ref|XP_002628671.1|  conserved hypothetical protein [Blastomyces ...  40.8    2.6  
gb|EEQ89475.1|  conserved hypothetical protein [Blastomyces derma...  40.8    2.7  
ref|XP_772881.1|  hypothetical protein CNBK2520 [Cryptococcus neo...  40.0    4.2  
ref|XP_567786.1|  hypothetical protein [Cryptococcus neoformans v...  39.7    5.6  
gb|KKZ65383.1|  hypothetical protein EMCG_08787 [Emmonsia crescen...  39.3    8.5  


 >emb|CED82152.1| hypothetical protein [Xanthophyllomyces dendrorhous]
Length=405

 Score =  764 bits (1972),  Expect = 0.0, Method: Compositional matrix adjust.
 Identities = 392/405 (97%), Positives = 392/405 (97%), Gaps = 12/405 (3%)

Query  1    MLLKAIGRPIRLSLVGKAYSVLPSLSTLTSGTLQDPKSSVLHVGSKSQASKK--------  52
            MLLKAIGRPIRLSLVGKAYSVLPSLSTLTSGTLQDPKSSVLHVGSKSQASKK        
Sbjct  1    MLLKAIGRPIRLSLVGKAYSVLPSLSTLTSGTLQDPKSSVLHVGSKSQASKKVYYKIGDK  60

Query  53   ----LNSYQLYTLYNPIKDSKIQFVDELSFGFTKHASNAKSSNFDRDALVETLSHQIETN  108
                LNSYQLYTLYNPIKDSKIQFVDELSFGFTKHASNAKSSNFDRDALVETLSHQIETN
Sbjct  61   DVSRLNSYQLYTLYNPIKDSKIQFVDELSFGFTKHASNAKSSNFDRDALVETLSHQIETN  120

Query  109  SSALSPLQLSSSLTKKYSDSILNLSLTATRRINKLWSVESKCWVPLSPPDDLYHRMDKFV  168
            SSALSPLQLSSSLTKKYSDSILNLSLTATRRINKLWSVESKCWVPLSPPDDLYHRMDKFV
Sbjct  121  SSALSPLQLSSSLTKKYSDSILNLSLTATRRINKLWSVESKCWVPLSPPDDLYHRMDKFV  180

Query  169  IGVVTASDLINGLNKDKRCLQKAVEELKAHWINQRDLVESDSSLSDKQLDSSNGQPIVVI  228
            IGVVTASDLINGLNKDKRCLQKAVEELKAHWINQRDLVESDSSLSDKQLDSSNGQPIVVI
Sbjct  181  IGVVTASDLINGLNKDKRCLQKAVEELKAHWINQRDLVESDSSLSDKQLDSSNGQPIVVI  240

Query  229  CLVGWGPAKLKMKADNQIAEAMDLETEILKTSIQEGCLISEAMNPKDAYDLIWSMLGDFA  288
            CLVGWGPAKLKMKADNQIAEAMDLETEILKTSIQEGCLISEAMNPKDAYDLIWSMLGDFA
Sbjct  241  CLVGWGPAKLKMKADNQIAEAMDLETEILKTSIQEGCLISEAMNPKDAYDLIWSMLGDFA  300

Query  289  RKVQVDNERQSFIHTDPDLVVPKGKSQEKAYSLMLQQIPSISAANVKAITSAYPTLRVLM  348
            RKVQVDNERQSFIHTDPDLVVPKGKSQEKAYSLMLQQIPSISAANVKAITSAYPTLRVLM
Sbjct  301  RKVQVDNERQSFIHTDPDLVVPKGKSQEKAYSLMLQQIPSISAANVKAITSAYPTLRVLM  360

Query  349  EALSTEAGFEVPKFPSEKAALIRHALTTEDPNSIIDAPKSKVQVA  393
            EALSTEAGFEVPKFPSEKAALIR ALTTEDPNSIIDAPKSKVQVA
Sbjct  361  EALSTEAGFEVPKFPSEKAALIRRALTTEDPNSIIDAPKSKVQVA  405


>emb|CDZ96279.1| EME1, MMS4 [Xanthophyllomyces dendrorhous]
Length=824

 Score = 50.8 bits (120),  Expect = 0.002, Method: Compositional matrix adjust.
 Identities = 31/100 (31%), Positives = 48/100 (48%), Gaps = 3/100 (3%)

Query  252  LETEILKTSIQEGCLISEAMNPKDAYDLIWSMLGDFA-RKVQVDNERQSFIHTDPDLVVP  310
            +E  +++  I E C +  +       + I S +GD A R  ++  +     H  PD    
Sbjct  669  IEKALVRLQIAESCFLMHSETDSQMLEGIISTVGDVAIRPYKLIEKTHLSFH--PDKSFR  726

Query  311  KGKSQEKAYSLMLQQIPSISAANVKAITSAYPTLRVLMEA  350
            +G S    Y  ML+QIP ++ A   A+   YPTLR LM+A
Sbjct  727  RGSSFGDTYRKMLEQIPRVTPATSSAVVEEYPTLRTLMDA  766


>ref|XP_007351565.1| hypothetical protein AURDEDRAFT_170588 [Auricularia delicata 
TFB-10046 SS5]
 gb|EJD40407.1| hypothetical protein AURDEDRAFT_170588 [Auricularia delicata 
TFB-10046 SS5]
Length=464

 Score = 50.1 bits (118),  Expect = 0.003, Method: Compositional matrix adjust.
 Identities = 35/135 (26%), Positives = 63/135 (47%), Gaps = 6/135 (4%)

Query  217  LDSSNGQPIVVICLVGWGPAKLKMKADNQIAEAMDLETEILKTSIQEGCLISEAMNPKDA  276
            L ++  +P   + L+  G  ++K +A     E +    +IL     + C +    + +DA
Sbjct  287  LAAARSRPNDQVFLMVHGLRQVKDRATRDKVEGILARVQIL-----QKCFMVHVTDARDA  341

Query  277  YDLIWSMLGDFARKVQVDNERQSFIHTDPDLVVPKGKSQEKAYSLMLQQIPSISAANVKA  336
             + I ++ GD A +     E      T PDLV   G+ + + Y  ML +IP  +  N ++
Sbjct  342  AERIINITGDLAVRPYKIIEMTHLPFT-PDLVKKCGRGERETYVHMLAKIPHFTEKNARS  400

Query  337  ITSAYPTLRVLMEAL  351
            + +A+PT   L  AL
Sbjct  401  VAAAFPTFDALFSAL  415


>gb|KIR87918.1| crossover junction endonuclease EME1 [Cryptococcus gattii IND107]
Length=481

 Score = 48.1 bits (113),  Expect = 0.010, Method: Compositional matrix adjust.
 Identities = 57/249 (23%), Positives = 104/249 (42%), Gaps = 32/249 (13%)

Query  126  SDSILNLSLTATRRINKLWSVESKCWVPLSPPDDLYHRMDKFVIGVVTASDLINGLNKDK  185
            SDS ++  +   R +   W  ESK +VPL  P  ++   +K V+ ++TA +L++      
Sbjct  193  SDSPISGVIRFKRYLQARWDPESKRFVPLDDPRWVW---EKTVLVIITAEELVD------  243

Query  186  RCLQKAVEELKAHWINQRDLVESDSSLSDKQL-------------------DSSNGQPIV  226
            +  +   ++L A W++   L+    ++SD+ +                   D  N     
Sbjct  244  KIAEGGEKDLLAQWVSDVRLLLG-LAMSDQMVLMIKGLQKYYSKSRALANKDYMNAARAG  302

Query  227  VICLVGWGPAKLKMKADNQIAEAMDLETEILKTSIQEGCLISEAMNPKDAYDLIWSMLGD  286
            +    G G    K    N       +E E+++  + E C +      +D  D ++++  D
Sbjct  303  LEGGSGSGSTATKTARINGRRTRDRIEAEMVRLQVAEHCFLVHVEKTQDMEDWVYNIAAD  362

Query  287  FA-RKVQVDNERQSFIHTDPDLVVPKGKSQEKAYSLMLQQIPSISAANVKAITSAYPTLR  345
             A R  ++ +  +S ++  P     K         LMLQ++  I+ +    IT  YPT R
Sbjct  363  IALRPYKLIS--KSHLNFAPAEGSKKALQTTAVLELMLQEVQGITPSAAAGITEKYPTFR  420

Query  346  VLMEALSTE  354
             LMEA   E
Sbjct  421  RLMEAFEME  429


>gb|KIR63608.1| crossover junction endonuclease EME1 [Cryptococcus gattii CA1873]
Length=487

 Score = 48.1 bits (113),  Expect = 0.012, Method: Compositional matrix adjust.
 Identities = 57/250 (23%), Positives = 102/250 (41%), Gaps = 31/250 (12%)

Query  126  SDSILNLSLTATRRINKLWSVESKCWVPLSPPDDLYHRMDKFVIGVVTASDLINGLNKDK  185
            SDS +   +   R +   W  ESK +VPL  P  ++   +K V+ ++TA +L+     DK
Sbjct  196  SDSPIPGVIRFKRYLQARWDPESKRFVPLDDPRWVW---EKTVLVIITAEELV-----DK  247

Query  186  RCLQKAVEELKAHWINQRDLV--------------------ESDSSLSDKQLDSSNGQPI  225
                   +++ A W++   L+                        +L++K   ++    +
Sbjct  248  IAEGGGEKDVLAQWVSDVRLLLGLTMSDQVVLVIKGLQKYYSKSRALANKDYINAARAGL  307

Query  226  VVICLVGWGPAKLKMKADNQIAEAMDLETEILKTSIQEGCLISEAMNPKDAYDLIWSMLG  285
                  G G    K    N       +E E+++  + E C +      +D  D ++++ G
Sbjct  308  EGGSGSGPGSTATKTARINGRPTRDRIEAEMVRLQVAEHCFLVHVEKTQDVEDWVYNIAG  367

Query  286  DFA-RKVQVDNERQSFIHTDPDLVVPKGKSQEKAYSLMLQQIPSISAANVKAITSAYPTL  344
            D A R  ++ +  +S ++  P     K         LMLQ++  I+ +    IT  YPT 
Sbjct  368  DIALRPYKLIS--KSHLNFAPAEGSKKALQPTAVLELMLQEVHGITPSAAAGITEKYPTF  425

Query  345  RVLMEALSTE  354
            R LMEA   E
Sbjct  426  RRLMEAFEME  435


>gb|KIR47882.1| crossover junction endonuclease EME1 [Cryptococcus gattii CA1280]
Length=495

 Score = 48.1 bits (113),  Expect = 0.013, Method: Compositional matrix adjust.
 Identities = 29/104 (28%), Positives = 50/104 (48%), Gaps = 3/104 (3%)

Query  252  LETEILKTSIQEGCLISEAMNPKDAYDLIWSMLGDFA-RKVQVDNERQSFIHTDPDLVVP  310
            +E E+++  + E C +      +D  D ++++ GD A R  ++ +  +S ++  P     
Sbjct  342  IEAEMVRLQVAEHCFLVHVEKTQDVEDWVYNIAGDIALRPYKLIS--KSHLNFAPAEGSK  399

Query  311  KGKSQEKAYSLMLQQIPSISAANVKAITSAYPTLRVLMEALSTE  354
            K         LMLQ++  I+ +    IT  YPT R LMEA   E
Sbjct  400  KALQPTAVLELMLQEVHGITPSAAAGITEKYPTFRRLMEAFEME  443


>gb|KEP53600.1| ERCC4 domain protein [Rhizoctonia solani 123E]
Length=456

 Score = 47.8 bits (112),  Expect = 0.015, Method: Compositional matrix adjust.
 Identities = 45/161 (28%), Positives = 71/161 (44%), Gaps = 21/161 (13%)

Query  202  QRDLVESDSSLSDKQLDSSNGQPIVVI-CLVGWGPAKLKMKAD-NQIAEAMDLETEILKT  259
            Q D+ E  +SL      +++ Q  ++I  L G+   K   K + N I  A+        T
Sbjct  276  QEDITEMLASLRRTHCLTTHSQIFIMIDGLNGYYKRKKGRKDERNTIESAL--------T  327

Query  260  SIQ--EGCLISEAMNPKDAYDLIWSMLGDFA----RKVQVDNERQSFIHTDPDLVVPKGK  313
            S+Q  E C I      +D    ++++ GD      R++Q     +SF+    D  V  G 
Sbjct  328  SLQALERCFIVHVEGAEDTAQWLFNITGDLGIRPHRRIQ-----ESFLPFCTDTQVKCGS  382

Query  314  SQEKAYSLMLQQIPSISAANVKAITSAYPTLRVLMEALSTE  354
            S+   Y  MLQQI +++ +    I    PTLR L E  + E
Sbjct  383  SKADTYKKMLQQIRNVTESAADGIIQEAPTLRELFEGYARE  423


>gb|EMS25983.1| ERCC4 domain containing protein [Rhodosporidium toruloides NP11]
 emb|CDR38351.1| RHTO0S03e08548g1_1 [Rhodosporidium toruloides]
Length=665

 Score = 47.8 bits (112),  Expect = 0.015, Method: Compositional matrix adjust.
 Identities = 30/102 (29%), Positives = 50/102 (49%), Gaps = 3/102 (3%)

Query  251  DLETEILKTSIQEGCLISEAMNPKDAYDLIWSMLGDFARK--VQVDNERQSFIHTDPDLV  308
            DLE E+++  ++  C+I       +A D    +  D  +K   ++ +   S + T  D V
Sbjct  512  DLELELMRVQVKSRCMIVSVDKVAEAVDWFEQLTFDIGQKPYQRLKHSHISILGTSEDKV  571

Query  309  VPKGKSQEKAYSLMLQQIPSISAANVKAITSAYPTLRVLMEA  350
            V  GK  +  Y  ML  +P ++ +  K I + YPTLR L E+
Sbjct  572  V-SGKDLQDTYIRMLASLPKVTESVAKGIVAEYPTLRALYES  612


>gb|EUC67094.1| ERCC4 domain protein, partial [Rhizoctonia solani AG-3 Rhs1AP]
Length=414

 Score = 47.4 bits (111),  Expect = 0.020, Method: Compositional matrix adjust.
 Identities = 45/161 (28%), Positives = 71/161 (44%), Gaps = 21/161 (13%)

Query  202  QRDLVESDSSLSDKQLDSSNGQPIVVI-CLVGWGPAKLKMKAD-NQIAEAMDLETEILKT  259
            Q D+ E  +SL      +++ Q  ++I  L G+   K   K + N I  A+        T
Sbjct  218  QEDITEMLASLRRTHCLTTHSQIFIMIDGLNGYYKRKKGRKDERNTIESAL--------T  269

Query  260  SIQ--EGCLISEAMNPKDAYDLIWSMLGDFA----RKVQVDNERQSFIHTDPDLVVPKGK  313
            S+Q  E C I      +D    ++++ GD      R++Q     +SF+    D  V  G 
Sbjct  270  SLQALERCFIVHVEGAEDTAQWLFNITGDLGIRPHRRIQ-----ESFLPFCTDTQVKCGS  324

Query  314  SQEKAYSLMLQQIPSISAANVKAITSAYPTLRVLMEALSTE  354
            S+   Y  MLQQI +++ +    I    PTLR L E  + E
Sbjct  325  SKADTYKKMLQQIRNVTESAADGIIQEAPTLRELFEGYARE  365


>gb|KDN49392.1| hypothetical protein RSAG8_02094, partial [Rhizoctonia solani 
AG-8 WAC10335]
Length=470

 Score = 47.4 bits (111),  Expect = 0.022, Method: Compositional matrix adjust.
 Identities = 32/98 (33%), Positives = 46/98 (47%), Gaps = 3/98 (3%)

Query  259  TSIQ--EGCLISEAMNPKDAYDLIWSMLGDFARKVQVDNERQSFIHTDPDLVVPKGKSQE  316
            TS+Q  E C I      +DA   ++++ GD   +      R+SF+    D  V  G S+ 
Sbjct  332  TSLQALERCFIVHVEGAEDAAQWLYNITGDLGIRPH-KRIRESFLPFCTDTQVKCGSSKA  390

Query  317  KAYSLMLQQIPSISAANVKAITSAYPTLRVLMEALSTE  354
              Y  MLQQI +I+ +    I    PTLR L E  + E
Sbjct  391  DTYKKMLQQIRNITESAADGIIEEAPTLRELFEGYARE  428


>gb|EKD04587.1| vesicle membrane protein (v-SNARE) with acyltransferase activity, 
Ykt6p [Trichosporon asahii var. asahii CBS 8904]
Length=816

 Score = 45.4 bits (106),  Expect = 0.091, Method: Compositional matrix adjust.
 Identities = 33/108 (31%), Positives = 54/108 (50%), Gaps = 7/108 (6%)

Query  248  EAMDLETEILKTSIQEGCLISEAMNPKDAYDLIWSMLGDFARKVQVDNERQSFIHTDPDL  307
            E   +E E+L+  + +  L+ +    +D  D I+++ GD A K  ++     F  + PD 
Sbjct  668  EKQQVEAELLQAQLSQYLLVVQ----EDIEDWIFNLAGDIAAKPLLEKSHLPF--SAPDH  721

Query  308  VVPKGKSQEKAYSLMLQQIPSISAANVKAITSAYPTLRVLMEALSTEA  355
               K      A  LMLQ++  ++A+    IT+ YP+ R LMEA   EA
Sbjct  722  PR-KAVDAMPALELMLQEVNGLTASAASGITTRYPSFRKLMEAYEGEA  768


>gb|KIK71135.1| hypothetical protein GYMLUDRAFT_235500 [Gymnopus luxurians FD-317 
M1]
Length=575

 Score = 45.4 bits (106),  Expect = 0.098, Method: Compositional matrix adjust.
 Identities = 49/237 (21%), Positives = 101/237 (43%), Gaps = 36/237 (15%)

Query  122  TKKYSDSILNLSLTA--TRRINKLWSVESKCWVPLSPPDDLYHRMDKFVIGVVTASDLIN  179
            T KY  S+L    T    RRI++ ++  S+ W+P+ PP   + R+    + +     L+N
Sbjct  317  TIKYGPSLLPGYDTVRWERRIHREYT--SRKWLPVDPPYLKFERLALIRLAINQLRTLVN  374

Query  180  GLNKDKRCLQKAVEELKAHWINQRDLVESDSSLSDKQLDSSNG-QPIVVICLVGWGPAKL  238
                         E    H++                L  ++G  P+  + ++ +G  +L
Sbjct  375  -------------ENKLGHFVGH--------------LRETHGLGPVDQVFIMVFGMGRL  407

Query  239  KMKADNQIAEAMDLETEILKTSIQEGCLISEAMNPKDAYDLIWSMLGDFARKVQVDNERQ  298
            + +    + E    E  +     +E    +   + ++A D +++  GD   + +    R 
Sbjct  408  RKR---NLQEWNKYEDALTSLQFREHTYHAYVEDDQEAVDRLYNYSGDLGIREEKLISR-  463

Query  299  SFIHTDPDLVVPKGKSQEKAYSLMLQQIPSISAANVKAITSAYPTLRVLMEALSTEA  355
            S++    ++ + KGK  +  ++LML +I  ++ +  K I + YPT+R L  A   +A
Sbjct  464  SYLPFCSNVKMEKGKDYQDIWTLMLGEIHRVTDSGAKGIVAEYPTMRTLRIAYDGQA  520


>gb|KIR54999.1| crossover junction endonuclease EME1 [Cryptococcus gattii Ru294]
Length=495

 Score = 45.1 bits (105),  Expect = 0.12, Method: Compositional matrix adjust.
 Identities = 28/104 (27%), Positives = 49/104 (47%), Gaps = 3/104 (3%)

Query  252  LETEILKTSIQEGCLISEAMNPKDAYDLIWSMLGDFA-RKVQVDNERQSFIHTDPDLVVP  310
            +E E+++  + E C +      +D  D ++++  D A R  ++ +  +S ++  P     
Sbjct  342  IEAEMVRLQVAEHCFLVHVEKTQDVEDWVYNIAADIALRPYKLIS--KSHLNFAPAEGSK  399

Query  311  KGKSQEKAYSLMLQQIPSISAANVKAITSAYPTLRVLMEALSTE  354
            K         LMLQ++  I+ +    IT  YPT R LMEA   E
Sbjct  400  KALQPTAVLELMLQKVQGITPSAAAGITEKYPTFRRLMEAFEME  443


>ref|XP_003196799.1| hypothetical protein CGB_K3270C [Cryptococcus gattii WM276]
 gb|ADV25012.1| Hypothetical Protein CGB_K3270C [Cryptococcus gattii WM276]
 gb|KIR79125.1| crossover junction endonuclease EME1 [Cryptococcus gattii EJB2]
 gb|KJE05853.1| crossover junction endonuclease EME1 [Cryptococcus gattii NT-10]
Length=493

 Score = 45.1 bits (105),  Expect = 0.12, Method: Compositional matrix adjust.
 Identities = 28/104 (27%), Positives = 49/104 (47%), Gaps = 3/104 (3%)

Query  252  LETEILKTSIQEGCLISEAMNPKDAYDLIWSMLGDFA-RKVQVDNERQSFIHTDPDLVVP  310
            +E E+++  + E C +      +D  D ++++  D A R  ++ +  +S ++  P     
Sbjct  340  IEAEMVRLQVAEHCFLVHVEKTQDVEDWVYNIAADIALRPYKLIS--KSHLNFAPAEGSK  397

Query  311  KGKSQEKAYSLMLQQIPSISAANVKAITSAYPTLRVLMEALSTE  354
            K         LMLQ++  I+ +    IT  YPT R LMEA   E
Sbjct  398  KALQPTAVLELMLQKVQGITPSAAAGITEKYPTFRRLMEAFEME  441


>gb|EJT46446.1| vesicle membrane protein (v-SNARE) with acyltransferase activity, 
Ykt6p [Trichosporon asahii var. asahii CBS 2479]
Length=816

 Score = 45.1 bits (105),  Expect = 0.14, Method: Compositional matrix adjust.
 Identities = 33/108 (31%), Positives = 54/108 (50%), Gaps = 7/108 (6%)

Query  248  EAMDLETEILKTSIQEGCLISEAMNPKDAYDLIWSMLGDFARKVQVDNERQSFIHTDPDL  307
            E   +E E+L+  + +  L+ +    +D  D I+++ GD A K  ++     F  + PD 
Sbjct  668  EKEQVEAELLQAQLSQYLLVVQ----EDIEDWIFNLAGDIAAKPLLEKSHLPF--SAPDH  721

Query  308  VVPKGKSQEKAYSLMLQQIPSISAANVKAITSAYPTLRVLMEALSTEA  355
               K      A  LMLQ++  ++A+    IT+ YP+ R LMEA   EA
Sbjct  722  PR-KAVDAMPALELMLQEVNGLTASAASGITTRYPSFRKLMEAYEGEA  768


>gb|KIY35389.1| crossover junction endonuclease EME1 [Cryptococcus gattii E566]
Length=493

 Score = 44.3 bits (103),  Expect = 0.17, Method: Compositional matrix adjust.
 Identities = 28/104 (27%), Positives = 49/104 (47%), Gaps = 3/104 (3%)

Query  252  LETEILKTSIQEGCLISEAMNPKDAYDLIWSMLGDFA-RKVQVDNERQSFIHTDPDLVVP  310
            +E E+++  + E C +      +D  D ++++  D A R  ++ +  +S ++  P     
Sbjct  340  IEAEMVRLQVAEYCFLVHVEKTQDVEDWVYNIAADIALRPYKLIS--KSHLNFAPAEGSK  397

Query  311  KGKSQEKAYSLMLQQIPSISAANVKAITSAYPTLRVLMEALSTE  354
            K         LMLQ++  I+ +    IT  YPT R LMEA   E
Sbjct  398  KALQPTAVLELMLQKVQGITPSAAAGITEKYPTFRRLMEAFEME  441


>ref|XP_001731355.1| hypothetical protein MGL_1538 [Malassezia globosa CBS 7966]
 gb|EDP44141.1| hypothetical protein MGL_1538 [Malassezia globosa CBS 7966]
Length=316

 Score = 43.9 bits (102),  Expect = 0.21, Method: Compositional matrix adjust.
 Identities = 32/110 (29%), Positives = 53/110 (48%), Gaps = 9/110 (8%)

Query  243  DNQIAEAMDLETEILKTSIQEGCLISEAMNPKDAYDLIWSMLGDFARKVQVDNERQSFIH  302
            D+Q  E M  E  +L+  ++  C +      ++A + ++S+  D + +     E +S  H
Sbjct  166  DDQTREHM--EQRLLQLQLRHQCHVVRVTQAEEAVEWVYSIACDISIRPYKQLESESVYH  223

Query  303  TDPDLVVPKGKSQEK--AYSLMLQQIPSISAANVKAITSAYPTLRVLMEA  350
                  V K  S     AY  ML++IP  ++    AIT+ YPTL  LM+A
Sbjct  224  K-----VNKAASSNSFSAYRAMLEEIPRCTSNASAAITAKYPTLASLMQA  268


>ref|XP_012048427.1| crossover junction endonuclease EME1 [Cryptococcus neoformans 
var. grubii H99]
 gb|AGV14193.1| crossover junction endonuclease EME1 [Cryptococcus neoformans 
var. grubii H99]
Length=601

 Score = 43.1 bits (100),  Expect = 0.48, Method: Compositional matrix adjust.
 Identities = 28/104 (27%), Positives = 49/104 (47%), Gaps = 3/104 (3%)

Query  252  LETEILKTSIQEGCLISEAMNPKDAYDLIWSMLGDFA-RKVQVDNERQSFIHTDPDLVVP  310
            +E E+++  + E   +      +D  D ++++  D A R  ++ +  +S ++  P     
Sbjct  448  IEAEMVRLQVAEHFFLVHVEKTEDVEDWVYNIAADIALRPYKLVS--KSHLNFAPAEGTR  505

Query  311  KGKSQEKAYSLMLQQIPSISAANVKAITSAYPTLRVLMEALSTE  354
            K         LMLQ++  I+A+    IT  YPT R LMEA   E
Sbjct  506  KALQPTAVLELMLQEVQGITASAAAGITEKYPTFRRLMEAFEME  549


>ref|XP_013240836.1| hypothetical protein K437DRAFT_276193 [Tilletiaria anomala UBC 
951]
 gb|KDN38796.1| hypothetical protein K437DRAFT_276193 [Tilletiaria anomala UBC 
951]
Length=680

 Score = 41.6 bits (96),  Expect = 1.5, Method: Compositional matrix adjust.
 Identities = 27/105 (26%), Positives = 51/105 (49%), Gaps = 3/105 (3%)

Query  252  LETEILKTSIQEGCLISEAMNPKDAYDLIWSMLGDFA-RKVQVDNERQSFIHTDPDLVVP  310
            L+ E++K  + E C + +     +    I S++ D   R  ++   R S +    D  V 
Sbjct  518  LDRELMKLRVAERCFVVQVEKSGEVVQWIVSIIQDVGIRPYKLI--RNSHLSFAVDTKVA  575

Query  311  KGKSQEKAYSLMLQQIPSISAANVKAITSAYPTLRVLMEALSTEA  355
             G + E  +S MLQQIP ++     +I   + T+R LM+++ ++ 
Sbjct  576  AGLTDEATFSAMLQQIPRMTEPQADSIVYTFATMRNLMDSIHSKG  620


>ref|XP_013084856.1| PREDICTED: crossover junction endonuclease MUS81-like [Biomphalaria 
glabrata]
Length=276

 Score = 40.0 bits (92),  Expect = 2.6, Method: Compositional matrix adjust.
 Identities = 25/48 (52%), Positives = 26/48 (54%), Gaps = 6/48 (13%)

Query  323  LQQIPSISAANVKAITSAYPTLRVLMEALSTEAGFEVPKFPSEKAALI  370
            L QIP ISA   KAIT  YPTL  LMEA      FE  K   EK  L+
Sbjct  204  LFQIPGISADRAKAITRVYPTLSFLMEA------FENCKDAKEKEKLL  245


>ref|XP_002628671.1| conserved hypothetical protein [Blastomyces dermatitidis SLH14081]
 gb|EEQ73349.1| conserved hypothetical protein [Blastomyces dermatitidis SLH14081]
Length=631

 Score = 40.8 bits (94),  Expect = 2.6, Method: Compositional matrix adjust.
 Identities = 30/110 (27%), Positives = 47/110 (43%), Gaps = 10/110 (9%)

Query  252  LETEILKTSIQEGCLISEAMNPKDAYDLIWSMLGDFARKVQVDNERQSFIH-TDPDLV--  308
            +E  +L+  +Q GCLI     P ++ + I     +F   V     RQ  I+  D      
Sbjct  482  IEDALLQLQVQHGCLIHHTTAPSESAEWI----KNFTEHVSTIPYRQELINLHDASFCMD  537

Query  309  ---VPKGKSQEKAYSLMLQQIPSISAANVKAITSAYPTLRVLMEALSTEA  355
               V  G+     Y  MLQ++  ++A     ITS YPT+  L+  L  + 
Sbjct  538  VGQVKSGEDNRDTYVKMLQEVQRVTAPISYGITSQYPTVAELVRQLKEQG  587


>gb|EEQ89475.1| conserved hypothetical protein [Blastomyces dermatitidis ER-3]
 gb|EGE79404.1| hypothetical protein BDDG_02343 [Blastomyces dermatitidis ATCC 
18188]
 gb|EQL31585.1| hypothetical protein BDFG_06047 [Blastomyces dermatitidis ATCC 
26199]
Length=631

 Score = 40.8 bits (94),  Expect = 2.7, Method: Compositional matrix adjust.
 Identities = 30/110 (27%), Positives = 47/110 (43%), Gaps = 10/110 (9%)

Query  252  LETEILKTSIQEGCLISEAMNPKDAYDLIWSMLGDFARKVQVDNERQSFIH-TDPDLV--  308
            +E  +L+  +Q GCLI     P ++ + I     +F   V     RQ  I+  D      
Sbjct  482  IEDALLQLQVQHGCLIHHTTAPSESAEWI----KNFTEHVSTIPYRQELINLHDASFCMD  537

Query  309  ---VPKGKSQEKAYSLMLQQIPSISAANVKAITSAYPTLRVLMEALSTEA  355
               V  G+     Y  MLQ++  ++A     ITS YPT+  L+  L  + 
Sbjct  538  VGQVKSGEDNRDTYVKMLQEVQRVTAPISYGITSQYPTVAELVRQLKEQG  587


>ref|XP_772881.1| hypothetical protein CNBK2520 [Cryptococcus neoformans var. neoformans 
B-3501A]
 gb|EAL18234.1| hypothetical protein CNBK2520 [Cryptococcus neoformans var. neoformans 
B-3501A]
Length=406

 Score = 40.0 bits (92),  Expect = 4.2, Method: Compositional matrix adjust.
 Identities = 27/104 (26%), Positives = 47/104 (45%), Gaps = 3/104 (3%)

Query  252  LETEILKTSIQEGCLISEAMNPKDAYDLIWSMLGDFA-RKVQVDNERQSFIHTDPDLVVP  310
            +E E+++  + E   +      +D  D ++++  D A R  ++    +S ++  P     
Sbjct  253  IEAEMVRLQVAEHFFLVHVEKTEDVEDWVYNIAADIALRPYKLIA--KSHLNFAPAEGSR  310

Query  311  KGKSQEKAYSLMLQQIPSISAANVKAITSAYPTLRVLMEALSTE  354
            K         LMLQ++  I+ +    IT  YPT R LMEA   E
Sbjct  311  KALQPTAVLELMLQEVQGITPSAAAGITEKYPTFRRLMEAFEME  354


>ref|XP_567786.1| hypothetical protein [Cryptococcus neoformans var. neoformans 
JEC21]
 gb|AAW46269.1| expressed protein [Cryptococcus neoformans var. neoformans JEC21]
Length=467

 Score = 39.7 bits (91),  Expect = 5.6, Method: Compositional matrix adjust.
 Identities = 57/274 (21%), Positives = 104/274 (38%), Gaps = 46/274 (17%)

Query  101  LSHQIETNSSALSPLQLSSSLTKKYSDSILNLSLTATRRINKLWSVESKCWVPLSPPDDL  160
            + H+I+   SAL  L           DS +  ++   R +   W   SK +VPL  P  +
Sbjct  168  IRHRIQDCQSALCFL--------PEPDSPIPGAIRFKRHLQARWDPGSKRFVPLDDPRWV  219

Query  161  YHRMDKFVIGVVTASDLINGLNKDKRCLQK-------------------AVEELKAHWIN  201
            +   +K V+ ++TA +L++ +      L +                    ++ L+ ++  
Sbjct  220  W---EKTVLVLITAEELVDKVADGGDTLGQWASDVRLLLGLATGDQVVLMIKGLQKYYSK  276

Query  202  QRDLVESDSSLSDKQLDSSNGQPIVVICLVGWGPAKLKMKADNQIAEAMDLETEILKTSI  261
             R L   D  ++  +     G            P   +     QI      E E+++  +
Sbjct  277  SRSLANKDY-INAARAGLEGGSAATAT------PITFRRPTREQI------EAEMVRLQV  323

Query  262  QEGCLISEAMNPKDAYDLIWSMLGDFA-RKVQVDNERQSFIHTDPDLVVPKGKSQEKAYS  320
             E   +      +D  D ++++  D A R  ++    +S ++  P     K         
Sbjct  324  AEHFFLVHVEKTEDVEDWVYNIAADIALRPYKLIA--KSHLNFAPAEGSRKALQPTAVLE  381

Query  321  LMLQQIPSISAANVKAITSAYPTLRVLMEALSTE  354
            LMLQ++  I+ +    IT  YPT R LMEA   E
Sbjct  382  LMLQEVQGITPSAAAGITEKYPTFRRLMEAFEME  415


>gb|KKZ65383.1| hypothetical protein EMCG_08787 [Emmonsia crescens UAMH 3008]
Length=628

 Score = 39.3 bits (90),  Expect = 8.5, Method: Compositional matrix adjust.
 Identities = 28/110 (25%), Positives = 48/110 (44%), Gaps = 10/110 (9%)

Query  252  LETEILKTSIQEGCLISEAMNPKDAYDLIWSMLGDFARKVQVDNERQSFIH-TDPDLV--  308
            +E  +L+  ++ GCLI     P ++ +  W  + +F   +     RQ  I+  D      
Sbjct  479  IEDALLQLQVEHGCLIQHTAEPAESAE--W--IKNFTEHISTIPYRQELINLHDASFCMD  534

Query  309  ---VPKGKSQEKAYSLMLQQIPSISAANVKAITSAYPTLRVLMEALSTEA  355
               V  G+     Y  MLQ+I  ++A     IT+ YPT+  L+  L  + 
Sbjct  535  VGQVKSGEDNADTYVKMLQEIQRVTAPISYGITTQYPTVGTLVRELKEQG  584


Lambda      K        H        a         alpha
   0.315    0.129    0.363    0.792     4.96 

Gapped
Lambda      K        H        a         alpha    sigma
   0.267   0.0410    0.140     1.90     42.6     43.6 

Effective search space used: 3640627020344


  Database: nr
    Posted date:  Sep 23, 2015 12:05 AM
  Number of letters in database: 26,053,659,533
  Number of sequences in database:  71,551,133


Matrix: BLOSUM62
Gap Penalties: Existence: 11, Extension: 1
Neighboring words threshold: 11
Window for multiple hits: 40
```
